# Handbook of Bacteriological Diagnosis

**Published:** 1902-09

**Authors:** 


**Handbook of Bacteriological Diagnosis.** By W. D'ESTE EMERY, M.D. Pp. xi., 215. H. K. Lewis. 1902.

This book aims at supplying to the practitioner the means of making bacteriological examinations in such cases where they may be necessary for purposes of diagnosis. It also contains useful information with regard to the collection and examination of certain morbid materials for transmission to the laboratory, rules for the examination of the blood, for the detection of certain hair parasites, and the making of sections. The directions given are precise and clear, and are such as can be carried out with the minimum provision of laboratory accommodation. Here and there, however, procedures are described which, if followed out, would undoubtedly result in erroneous conclusions, as when in staining for tubercle bacilli the student is directed to keep the film in 20 per cent. sulphuric acid for "three or four minutes"; or when in performing Widal's test he is directed to make his typhoid emulsion with *water*. In the one case the probability is that the bacilli would be decolourised, and in the other the water mixed with the blood might give rise to a spurious clumping. We should, too, strongly advise the student not to follow the directions of the book and suck up with the mouth the contents of a malignant pustule into a pipette.

